# Effect of *Lactobacillus plantarum* C014 on Innate Immune Response and Disease Resistance against *Aeromonas hydrophila* in Hybrid Catfish

**DOI:** 10.1155/2013/392523

**Published:** 2013-12-21

**Authors:** Sureerat Butprom, Parichat Phumkhachorn, Pongsak Rattanachaikunsopon

**Affiliations:** Department of Biological Science, Faculty of Science, Ubon Ratchathani University, Ubon Ratchathani 34190, Thailand

## Abstract

A bacterial strain isolated from intestines of hybrid catfish (*Clarias gariepinus* Male × *Clarias macrocephalus* Female) exhibited an *in vitro* inhibitory effect on a fish pathogen, *Aeromonas hydrophila* TISTR 1321. By using the 16S rDNA sequence analysis, it was identified as *Lactobacillus plantarum* C014. To examine whether *L. plantarum* C014 had potential for use as an immunostimulant and biocontrol agent in hybrid catfish, the fish diet supplemented with *L. plantarum* C014 (10^7^ CFU/g diet) was prepared and used for the *in vivo* investigation of its effect on innate immune response and disease resistance of hybrid catfish. Two innate immune response parameters, phagocytic activity of blood leukocytes and plasma lysozyme activity, were significantly enhanced in the treated fish after 45 days of feeding. Feeding the fish with the *L. plantarum* C014 supplemented diet for 45 days before challenging them with *A. hydrophila* at the dose of LD_50_ could reduce the mortality rate of the fish from 50% (in control group) to 0% (in treated group). Based on its origin and beneficial effect on innate immune response and disease resistance, *L. plantarum* C014 may be a potential candidate for use as a natural and safe immunostimulant and biocontrol agent in hybrid catfish.

## 1. Introduction

Hybrid catfish (*Clarias gariepinus* Male × *Clarias macrocephalus* Female) has become one of the most important protein sources for Thai people because of its low cost, rapid growth, high availability, and high nutritional value. To match the increasing demand of hybrid catfish, the production of hybrid catfish in Thailand has dramatically increased every year for more than ten years. However, hybrid catfish grown in the intensive aquaculture are often exposed to stressful conditions which have a negative impact on growth and immunity of the cultured fish. Therefore, hybrid catfish in such environments usually has low growth rate and high tendency to develop diseases, especially bacterial infectious diseases. Among bacteria identified as pathogens in hybrid catfish aquaculture, *Aeromonas hydrophila* is considered to be one of the foremost economically important pathogens because its infection can lead to growth reduction and unmarketable appearance of infected fish [1].

Currently, bacterial infections in aquaculture, including *A. hydrophila*  infection, are mainly controlled by antibiotics. However, recently, the use of antibiotics in aquaculture has received considerable attention because their use can lead to the development of drug resistant bacteria, thereby reducing drug efficacy. Drug resistance in fish pathogens may also transfer to environmental and human pathogenic bacteria [2]. Moreover, residues of drugs in fish can be potentially risky to consumers and the environment [3]. Therefore, the demand for safer alternative approaches to control the infections has been increasing. One of the most promising methods is to strengthen the defense mechanisms of fish through prophylactic administration of natural immunostimulants. These agents are well known to increase resistance to infectious diseases by enhancing innate (nonspecific) immunity [4]. Among natural immunostimulants, lactic acid bacteria (LAB), especially from fish gastrointestinal tracts, have become potential candidates to replace antibiotics for controlling diseases in fish due to their generally recognized as safe (GRAS) status and participation as key components in fish immune response. The relationship between normal endogenous microbiota and the innate immune system of fish was reviewed by Gómez and Balcázar [5]. Several research works have successfully utilized LAB to enhance fish immunity [6–8] and disease resistance ability [9–14]. The beneficial properties of LAB have brought them to our attention as biocontrol agents against *A. hydrophila* in hybrid catfish.

To obtain a strain of LAB that is suitable for being used as a biocontrol agent in hybrid catfish aquaculture, this study was carried out to isolate a LAB strain from hybrid catfish intestinal tract and evaluate its influence on innate immune response and resistance to *A. hydrophila*  infection of the fish.

## 2. Materials and Methods

### 2.1. Isolation of LAB from Hybrid Catfish Intestines

The intestines were aseptically taken from healthy hybrid catfish (*Clarias gariepinus* Male × *Clarias macrocephalus* Female) cultured at Ubon Ratchathani Rajabhat University, Ubon Ratchathani, Thailand. They were cut into small pieces and mixed with de Man, Rogosa, and Sharpe (MRS) broth to obtain a final concentration of 0.1% (w/v). The mixture was incubated at 30°C for 24 h and then centrifuged at 3,000 ×g for 3 min to remove the fish intestinal residue. One hundred mL of the supernatant was spreading onto MRS Agar and incubated at 30°C for 24 h. Twenty-five isolated LAB colonies grown on the MRS agar were randomly selected for the determination of their antimicrobial activity against a fish pathogenic bacterium *A. hydrophila* TISTR 1321 obtained from the Institute of Scientific and Technological Research, Ministry of Science and Technology, Thailand.

### 2.2. Determination of Antimicrobial Activity

Lactic acid bacteria isolated from fish intestines were determined for their antimicrobial activity against the fish pathogen *A. hydrophila* by using agar spot test. Five *μ*L of the tested LAB cultures grown overnight in MRS broth were spotted onto MRS agar and incubated at 30°C for 24 h. Then 5 mL of the soft (0.6% agar) Tryptic Soy Agar (TSA) containing log phase cells of *A. hydrophila* (at the final concentration of 10^6^ CFU/mL) was poured over the agar, previously inoculated with the tested LAB. After incubation at 30°C for 24 h, the presence of inhibition zone around the inoculated LAB was examined.

### 2.3. Identification of LAB

The LAB strain showing antimicrobial activity against *A. hydrophila*  was identified by Gram staining, morphological observation under light microscope, standard biochemical tests, and 16S rDNA sequence analysis. The 16S rDNA sequence analysis was performed as follows. Bacterial genomic DNA used as template DNA for PCR amplification of 16S rDNA was extracted from the bacterium by using the Genomic DNA Extraction Kit (Real Biotech Corporation, Taipei, Taiwan) according to the manufacturer's recommendations. The PCR amplification of 16S rDNA was performed using two universal primers, fd1 (5′ AGAGTTTGATCCTGGCTCAG 3′) [15], and 1492r (5′ ACGGCTACCTTGTTACGACTT 3′) [16]. The cycling protocol used in this study was 97°C for 5 min followed by 30 cycles of 97°C for 45 s, 55°C for 45 s, and 72°C for 90 s. The PCR product was purified and sequenced by BioDesign Co., Ltd. (Pathumthani, Thailand). The sequence of the 16S rDNA was compared with the 16S rDNA sequences in the GenBank database using the NCBI Blast program.

### 2.4. Fish Preparation

Healthy hybrid catfish (*Clarias gariepinus* Male × *Clarias macrocephalus* Female) were obtained from local farms in Ubon Ratchathnai Province, Thailand. To acclimate to laboratory conditions, fish were maintained in 2 m × 2 m × 0.6 m cement ponds for two weeks prior to experiments. On the day of stocking, the fish were fed 3% body weight twice a day with the commercial pellet fish diet for small catfish (Charoen Pokphand Group, Bangkok, Thailand). Twenty-four h prior to the experiments, fish weighing 50 ± 1 g were transferred from the stocking ponds to 180 cm × 60 cm × 60 cm aquaria (20 fish per aquarium). Aquaria were cleaned every third day by siphoning off two-thirds of water and replacing it with fresh water.

### 2.5. Preparation of LAB Supplemented Fish Diet

The isolated LAB used in this experiment was *Lactobacillus plantarum* C014 due to its possession of antimicrobial activity. Its stock suspension was prepared by suspending the LAB cells collected from 24 h culture of the bacterium in MRS broth to obtain a concentration of 10^10^ CFU/mL.

To prepare the fish diet supplemented with *L. plantarum* C014 at the final concentration of 10^7^ CFU/g diet, the bacterial stock suspension was sprayed into the commercial pellet fish diet for small catfish slowly, mixing part by part in a drum mixer. The mixture was passed through a minced-meat processing machine, producing extruded strings, which were dried at 30°C for 24 h and then broken down to about 2 mm long pellets. The diet was transferred to plastic bags and stored in a freezer (−20°C) until used. The *L. plantarum* C014 supplemented fish diet preparation was repeated every two weeks. Viability of the incorporated *L. plantarum* C014 bacterial cells in the diet was assessed by bacterial isolation on *Lactobacillus*  selective agar (NEOGEN, Lansing, MI, USA). The isolated bacterial colonies grown on the medium were randomly selected to confirm their identities by using the API 50 CH system (BioMerieux Industry, Hazelwood, MO, USA). For the control diet, it was prepared using the same process as the *L. plantarum* C014 supplemented diet except adding the same amount of sterile physiological saline to the commercial pellet fish diet instead of *L. plantarum* C014 suspension.

### 2.6. Preparation of Leukocytes and Plasma

To study the effect of *L. plantarum* C014 supplemented fish diet on innate immunity of hybrid catfish, groups of 20 uninfected fish were fed the diets with and without *L. plantarum* C014 separately. Each feeding was performed in three replicates. Blood samples (4 fish at a time in each group) from caudal vein were collected at 0, 15, 30, 45, and 60 days after feeding and kept separately in heparin coated plastic tubes (BD, Franklin Lakes, NJ, USA). Individual fish was sampled only once to avoid the influence on the assays due to multiple bleeding and handling stress on the fish. Leukocytes and plasma were prepared from each blood sample by using EZ Lympho-Sep-Lymphocyte Separation Tubes (Biological Industries, Kibbutz Beit-Haemek, Israel). Plasma was stored at −20°C until used for analysis. Separated leukocytes were washed twice in Hanks Balanced Salt Solution (HBSS) (Sigma-Aldrich, St. Louis, MO, USA) and resuspended in the same solution to obtain the concentration of 10^7^ cells/mL. The obtained leukocytes and plasma were used for the determination of phagocytic activity and lysozyme activity, respectively. Both activities are parameters of innate immunity.

### 2.7. Phagocytic Activity Assay

Phagocytic activity of blood leukocytes was determined by using the method of Seeley et al. [17] with some modification. One mL of leukocyte suspension was mixed with 2 mL of the Congo red-stained *Saccharomyces cerevisiae* cell suspension (providing a yeast cell : leukocyte ratio of 20 : 1). The mixtures were incubated at room temperature for 1 h. Following incubation, 1 mL of ice-cold HBSS was added and 1 mL of histopaque (1.077) was injected into the bottom of each sample tube. The samples were centrifuge at 850 ×g for 5 min to separate leukocytes from yeast cells. Leukocytes were harvested and washed twice in HBSS. The cells were then resuspended in 1 mL trypsin-EDTA solution (5.0 g/L trypsin and 2.0 g/L EDTA, Sigma-Aldrich) and incubated at 37°C overnight. The absorbance of the samples was measured at 510 nm using trypsin-EDTA as a blank.

Congo red-stained *S. cerevisiae* cells were prepared as follows. Three mL of a 0.87% (w/v) Congo red in phosphate buffer saline (PBS) pH 7.2 was added to yeast cell suspension (1.5 g). After incubated at room temperature for 15 min, the mixture was thoroughly mixed with 7 mL of distilled water. The resulting mixture was autoclaved for 15 min, washed three times in HBSS, and stored at 4°C until use. Prior to use, the cell concentration of was adjusted to 10^8^ cells/mL in HBSS.

### 2.8. Lysozyme Activity Assay

Plasma lysozyme activity was measured using the method of Ellis [18] with some modifications. Briefly, a volume of 0.02% (w/v) suspension of *Micrococcus lysodeikticus* ATCC No. 4698 (Sigma-Aldrich) prepared in 0.05 M phosphate buffer (pH 6.2) was used as a substrate. Lyophilized hen egg white lysozyme (Sigma-Aldrich) was used as a standard. One hundred mL of standard solutions and fish plasma were added to a 3 mL suspension of *M. lysodeikticus*. After incubation for 5 min at 25°C, the absorbance of the mixtures was examined at the wavelength of 550 nm. The lysozyme activity of fish plasma was reported as mg/mL equivalent of hen egg white lysozyme activity.

### 2.9. Determination of Median Lethal Dose (LD_50_) of *A. hydrophila*

An overnight culture of *A. hydrophila* TISTR 1321 in TSB was centrifuged at 5,000 rpm for 10 minutes. Bacterial cells were washed twice with physiological saline (0.85% NaCl) and then resuspended in the same solution to obtain a bacterial suspension with the concentration of 10^8^ CFU/mL. The bacterial suspension was subjected to tenfold serial dilutions using the physical saline as a diluent and then used to challenge groups of 20 hybrid catfish. One hundred *μ*L of each dilution was injected intraperitoneally into each fish. For control, the same volume of physiological saline was used instead of the bacterial suspension. Each dilution trial was performed in three replicates. Mortalities were recorded daily for 2 weeks. Dead fish were removed from the aquaria daily. Livers and kidneys were aseptically streaked on Aeromonas Medium Base  (Oxoid Limited, Hampshire, UK), a selective medium for *A. hydrophila*. After incubation at 30°C for 24 h, colonies grown on the agar were confirmed to be *A. hydrophila* by using the API 20 NE test kit (BioMerieux Industry, Hazelwood, MO, USA). The LD_50_ value was calculated by the method described by Reed and Muench [19].

### 2.10. Test for Effect of *L. plantarum* C014 Supplemented Fish Diet on Disease Resistance

Groups of 20 uninfected hybrid catfish with three replicates per each were fed the diets with and without *L. plantarum*  C014 separately for 45 days. After that, fish were challenged intraperitoneally with 0.1 mL of *A. hydrophila* TISTR 1321 suspension at a dose causing 50% mortality (LD_50_). The diets were continued for 14 d after infection. The mortality in each group was recorded daily. Dead fish were removed from the aquaria daily, and their livers and kidneys were subjected to bacterial isolation on Aeromonas Medium Base. After incubation at 30°C for 24 h, the bacteria grown on the medium were identified by using the API 20 NE test kit.

### 2.11. Statistical Analysis

Analysis of variance (ANOVA) was performed using the general linear models procedure of Statistical Analysis System (SAS Institute, Cary, NC, USA). Duncan's new multiple range test was used to obtain pairwise comparisons among sample means. Evaluations were based on a 5% significance level (*P* < 0.05).

## 3. Results

Among hundreds of LAB colonies isolated from healthy hybrid catfish's intestines, 25 colonies were randomly selected to examine their antimicrobial activity against a fish pathogen, *A. hydrophila* TISTR 1321. By using agar spot test, of all 25 LAB isolates, only one isolate, designated as C014, exhibited inhibitory effect on *A. hydrophila*, indicated by its ability to produce a clear zone against the lawn of *A. hydrophila*.

According to the morphological and biochemical characterization, the LAB isolate C014 was Gram-positive, catalase-negative bacillus. It was unable to produce ammonia from arginine or carbon dioxide from glucose. It was found to grow at 15°C but not at 45°C. The molecular identification of the LAB isolate C014 was carried out by using 16S rDNA sequence analysis. The 16S rDNA amplified from the LAB using universal primers (fd1 and 1492r) comprised about 1,500 bp. When the sequence of this amplicon was compared to the known bacterial 16S rDNA sequences in GenBank database using Blast program, it showed 99% homology to 16S rDNA sequence of *Lactobacillus plantarum* WCFS1 (accession NR 075041.1). Thus, the LAB isolate C014 was designated as *Lactobacillus plantarum* C014.

*L. plantarum* C014 was selected for further *in vivo* evaluation of its effect on innate immune response (phagocytic activity and lysozyme activity) and resistance to *A. hydrophila* infection of hybrid catfish because it was the only isolated strain having antimicrobial activity against *A. hydrophila* TISTR 1321.

**Figure 1:**
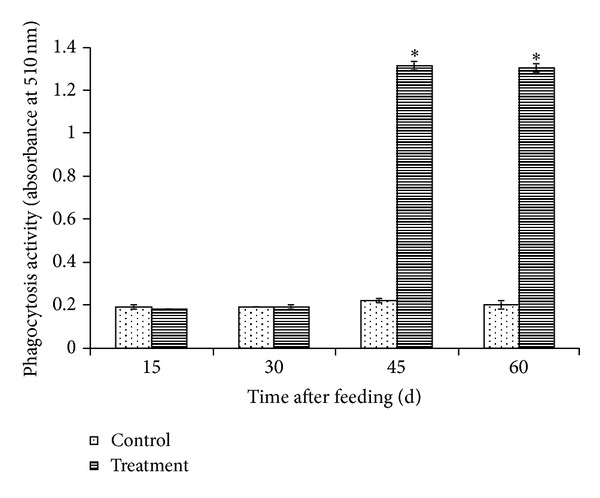
Phagocytic activity of isolated leukocytes of hybrid catfish fed diets with (treatment group) and without (control group) *L. plantarum*  C014. Results were presented as means ± SD of triplicate observations. Significant differences (*P* < 0.05) from the control group of the same day are indicated by asterisks.

In the first 30 days of feeding, no significant difference was observed between the phagocytic activity of leukocytes obtained from *L. plantarum* C014 treated fish and that obtained from the control fish. At day 45 of feeding, the increase of the phagocytic activity of leukocytes was detected in the treatment group but not in the control group. However, no further increase of the phagocytic activity of leukocytes was found in both treatment and control group after day 45 of feeding (Figure 1).

**Figure 2:**
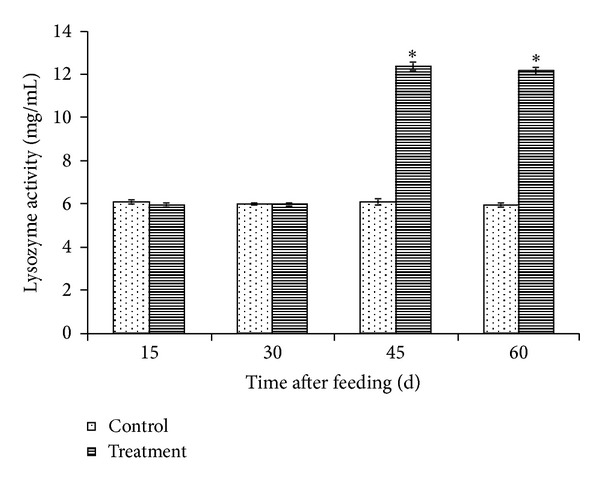
Lysozyme activity in hybrid catfish fed diets with (treatment group) and without (control group) *L. plantarum* C014. Results were presented as means ± SD of triplicate observations. Significant differences (*P* < 0.05) from the control group of the same day are indicated by asterisks.

The lysozyme activity of the treatment group was not significantly different from that of the control group for the first 30 days of feeding. However, the difference of this activity between the *L. plantarum* C014 treated and control fish was initially observed 45 days after feeding. Further feeding of *L. plantarum* C014 supplemented fish diet did not increase the lysozyme activity of the treatment group of fish (Figure 2).

Before we investigated the effect of *L. plantarum* C014 supplemented fish diet on resistance of hybrid catfish to the disease caused by *A. hydrophila*, the median lethal dose (LD_50_) of the pathogen for hybrid catfish was determined. The mortality of hybrid catfish in the first 2 weeks after intraperitoneal injection of different dilutions of *A. hydrophila* suspension is shown in Table 1. All of dead fish died within 5 days after bacterial injections and the pathogen was found in their livers and kidneys. Based on the mortality, the calculated LD_50_ of *A. hydrophila*  for catfish was 1.07 × 10^2^ CFU/g fish.

The effect of the diet supplemented with *L. plantarum* C014 on the resistance of hybrid catfish to *A. hydrophila*  infection was assessed by challenging the fish receiving the diet for 45 days with *A. hydrophila* at the dose of LD_50_. For the control group, the bacterial pathogen was given to the fish fed with the diet without *L. plantarum* C014 supplement. The results clearly showed that the *L. plantarum* C014 was able to prevent the fish from death caused by *A. hydrophila* indicated by the significant decrease of mortality rate from 50% (in the control fish) to 0% (in the *L. plantarum* C014 treated fish) (Table 2).

## 4. Discussion

In fish, the defense against invading pathogens relies mainly on the innate immune system [20]. It consists of 3 major elements including physical barriers, humoral and cellular components. Innate humoral components include antimicrobial peptides, lysozyme, complement, transferrin, pentraxins, lectins, antiproteases, and natural antibodies, whereas the predominant role of the innate cellular components is phagocytosis. Therefore, each of these components can be used as a parameter to determine status of innate immune response of fish. In this study, phagocytic activity and lysozyme activity were used to investigate the effect of *L. plantarum*  C014 supplemented fish diet on innate immune response of hybrid catfish. The reason that we selected oral administration over injection method to give *L. plantarum*  C014 to hybrid catfish is because oral administration is noninvasive and produces no stress to fish, compared to injection method. Furthermore, it is considered to be more practical in fish farming than injection.

Leukocytes are one of the most important cells of the innate immune system of fish. Their phagocytic activity is a primitive defense mechanism and important characteristic of the innate immune system [21]. Therefore, innate immune system and defense mechanism of fish can be enhanced by increasing phagocytic activity of fish leukocytes. In this study, the *L*. *plantarum* C014 was clearly shown to be a potential immunostimulant in hybrid catfish because fish diet supplemented with the bacterium boosted the phagocytic activity of fish leukocytes after feeding for 45 days. Several bacteria have been reported to be able to enhance phagocytic activity of fish leukocytes including *Bacillus circulans* PB7 [22], *Bacillus clausii* [23], *Bacillus pumilus* [23], *Bacillus subtilis* [7], and *Lactobacillus delbrueckii* subsp. *lactis* [7]. Currently, substantial amounts of research grants and efforts have been spent on studies to reveal how bacteria enhance the phagocytic activity in fish.

Lysozyme is a bactericidal protein hydrolyzing *β*-1,4-linked glycosidic bonds of bacterial cell wall peptidoglycans resulting in cell lysis. It is known to attack mainly Gram-positive bacteria as well as Gram-negative bacteria in conjunction with complements [20]. The *L. plantarum* C014 was shown in the present study to be a stimulant of lysozyme activity in hybrid catfish. Similar result was reported by Panigrahi et al. [6]. They found that the *Lactobacillus rhamnosus* JCM 1136 had stimulatory effect on lysozyme activity of rainbow trout *Oncorhynchus mykiss*. Many fish species including tilapia, catfish, rainbow trout have been known to use lysozyme as a part of innate immune system to protect themselves from pathogens. However, some fish such as cod, haddock, pollack, and wolffish have very little or no lysozyme activity in their tissues and body fluids. These fish on the other hand show high chitinase activity in their plasma and various organs [24]. Therefore, it is believed that these fish species may use chitinase instead of lysozyme to defend themselves against pathogens [20].

The *in vivo* study is imperative to investigate whether or not the *L. plantarum* C014 improve resistance of hybrid catfish to *A. hydrophila*  infection even though the bacterium was shown to have an inhibitory effect on *A. hydrophila* in the *in vitro* study. This is because several bacteria which were found to have antimicrobial activity *in vitro* did not improve disease resistance in fish. For example, Abd El-Rhman et al. [25] reported that  *Pseudomonas* sp. suppressed the growth of *Aeromonas salmonicida in vitro* but did not reduce the mortality, caused by  *A. salmonicida*  infection in Nile tilapia. The present study investigated the possibility of using *L. plantarum* C014 as a prophylactic agent to control *A. hydrophila*  infection in hybrid catfish. Since both innate immune response parameters used in this study (phagocytic and lysozyme activities) significantly increased after feeding the fish with *L. plantarum* C014 supplemented fish diet for 45 days, the fish were fed the diet for 45 days before challenging the fish with *A. hydrophila*. The cumulative mortality of *A. hydrophila* infected hybrid catfish fed the *L. plantarum* C014 containing fish diet decreases significantly, compared to that of the control group. Our results suggested that *L. plantarum* C014 not only inhibited *A. hydrophila in vitro* but enhanced resistance to the disease caused by *A. hydrophila* in hybrid catfish as well.

## 5. Conclusion

In conclusion, this study shows that *L. plantarum* C014 isolated from hybrid catfish's intestines when added to fish diet at the concentration of 10^7^ CFU/g diet improved innate immune response and disease resistance in hybrid catfish. The diet elevated not only phagocytic activity and lysozyme activity in hybrid catfish, but also the survival rate of *A. hydrophila*  infected hybrid catfish. These results suggest that *L. plantarum* C014 has a potential to be used as a natural and safe immunostimulants and biocontrol agent against  *A. hydrophila* in hybrid catfish. However, many interesting issues are still unclear and require further investigations for proper explanation. These include the following. How can *L. plantarum* C014 enhance fish innate immune response? Does the antimicrobial activity of *L. plantarum* C014 or the enhanced innate immunity of fish play an important role in the increase of disease resistance or both of them share an equal responsibility? Is the ability of *L. plantarum* C014 to raise innate immunity and disease resistance fish species specific and bacteria pathogen strain specific? Research works designed to address all of these questions are underway in our laboratory.

## Figures and Tables

**Table 1:** Mortality of hybrid catfish in the first 2 weeks after intraperitoneal injection of different dilutions of *A. hydrophila* suspension.

*A*. *hydrophila *		No. of dead fish/no. of tested fish^a^			% Mortality
Concentration (CFU/mL)	Dilution	1	2	3	
10^7^	10^−1^	17/20	18/20	17/20	86.67
10^6^	10^−2^	13/20	15/20	15/20	71.67
10^5^	10^−3^	10/20	11/20	12/20	55.00
10^4^	10^−4^	6/20	7/20	9/20	36.67
10^3^	10^−5^	4/20	3/20	4/20	18.37
10^2^	10^−6^	2/20	1/20	3/20	10.00

^a^results from all replicates (replicate 1 to replicate 3).

**Table 2:** Mortality of *A. hydrophila *infected hybrid catfish fed diets with and without *L. plantarum* C014.

Replicate	Mortality (%)	
	T	C
1	50	0
2	50	0
3	50	0

T: Treatment group fed the diet having *L. plantarum* C014.

C: Control group fed the diet without *L. plantarum* C014.
